# A review on One Health approach in Ethiopia

**DOI:** 10.1186/s42522-022-00064-z

**Published:** 2022-04-22

**Authors:** Gashaw Adane Erkyihun, Fikru Regassa Gari, Bedaso Mammo Edao, Gezahegne Mamo Kassa

**Affiliations:** 1 Ministry of Agriculture, Federal Democratic Republic of Ethiopia, P.O. Box 62347, Addis Ababa, Ethiopia; 2 College of Veterinary Medicine, Addis Ababa University, P.O. Box 34, Bishoftu, Ethiopia

**Keywords:** Awareness, Collaboration, Ethiopia, One Health Approach, Multi-sectoral, Zoonoses

## Abstract

The risk of spreading emerging and reemerging diseases has been increasing by the interactions of human – animal – ecosystems and increases account for more than one billion cases, a million deaths and caused hundreds of billions of US dollars of economic damage per year in the world. Countries in which their household income is dependent on livestock are characterized by a strong correlation between a high burden of zoonotic disease and poverty. The One Health approach is critical for solutions to prevent, prepare for, and respond to these complex threats. As part of the implementation of the Global Health Security Agenda, Ethiopia has embraced the One Health approach to respond to the existing and emerging threats. Several developments have been made to pioneer One Health schemes in Ethiopia which includes establishment of the National One Health Steering Committee and Technical Working Groups, prioritization of zoonotic diseases based on their impact on human and livestock, the development of prevention and control working documents for prioritized zoonotic diseases, joint disease surveillance and outbreak investigation, prioritization of zoonotic diseases**,** capacity building and other One Health promotions. Nevertheless, there are still so many challenges which need to be addressed. Poor integration among sectors in data sharing and communication, institutionalization of One Health, lack of continuous advocacy among the community, lack of financial funds from the government, limited research fund and activities on One Health, etc. are among many challenges. Hence, it is critical to continue raising awareness of One Health approach and foster leaders to work across disciplines and sectors. Therefore, continuous review on available global and national one health information and achievements to provide compiled information for more understanding is very important.

## Background

Zoonotic diseases account for more than one billion cases and a million deaths per year with the high costs responsible for emerging and pandemic ones [1]. Now a days, the risk of spreading these diseases has been increasing by the interactions of human – animal – ecosystem due to the exponential growth in human and livestock populations, rapid urbanization, rapidly changing farming systems, closer integration between livestock and wildlife with forest encroachment, destruction of habitat, changes in ecosystems, and the globalization of trade [2]. Nearly two-thirds of humans infectious diseases arise from pathogens shared with wild or domestic animals. However, ecological, evolutionary, social, economic, and epidemiological mechanisms affecting zoonoses persistence and emergence are not well understood. Multi-sectoral collaboration, including public health scientists, ecologists, veterinarians, economists, and others, is necessary for effective management of such diseases [3]. Health threats aggravators such as war, nutrition insecurity, pollution, loss of biodiversity, degraded ecosystem and climate change are becoming common factors [4]. A One Health approach is not only critical for solutions to respond to these threats but also an effective platform to address challenges, coordination mechanisms and global development goals [5]. Countries in which their household income depends on livestock are characterized by a strong correlation between a high burden of zoonotic disease and poverty (6). Ethiopia has the largest livestock, second largest human population and considerable wildlife species in Africa. About 80% of her citizens are dependent on agriculture and have direct contact with domestic animals. This could cause a high risk of zoonotic disease transmission and emerging and reemerging pandemic threats and the country is believed to be on a high burden of zoonotic disease [7]. The country is also near to East African countries which are frequently prone to emerging and epidemic diseases (such as avian influenza and Ebola). For instance, Ebola Virus Disease (EVD) outbreak has occurred more than 13 times, 6 times and 3 times in Democratic Republic Congo, Sudan and Uganda respectively [8]. On the other hand, a highly pathogenic avian influenza outbreak was reported from Uganda in 2017 and Zambia in 2019 [9]. In addition to these, the presence of endemic zoonotic diseases (like rabies, anthrax, brucellosis etc.) coupled with limited animal and human health care is also cause a significant impact on the national economy.Thus, reducing the zoonotic disease burden through One Health approach will improve the overall health of populations and contribute to the alleviation of poverty [10].

Ethiopia has achieved considerable One Health approach activities to push forward the Global Health Security Agenda (GHSA) commitments and to prevent, detect, and respond to existing and emerging threats since the 2000s. It has already established a National One Health Steering Committee (NOHSC) and Technical Working Groups (TWG) with a five-year strategic plan for the period 2018–2022. In Ethiopia, several achievements have been recorded so far (such as extension of one health schemes to the regional governments, joint disease surveillance and outbreak investigation activities, joint vaccination activities against zoonotic diseases, prioritization of zoonotic diseases, development of control and prevention strategic documents for different prioritized zoonotic diseases [11], one health and world rabies day celebration. However, awareness creation about One Health principles and importance, for the community and responsible bodies is limited. Little or no review (to provide compiled information) has been conducted regarding One Health in Ethiopia. Therefore, there is a need to strengthen One Health approach by reviewing available achievements, initiatives, activities and challenges. So, this review is believed to highlight potential areas of collaboration between the Ethiopian medical, veterinary sector and other scientific communities. A semi-quantitative method of review on available literature and consultations with selected One Health stakeholders was conducted. Specifically, all available information on One Health related to Ethiopia was searched for in global peer-reviewed databases using relevant search terms related to One Health. References are reviewed from retrieved articles to identify relevant publications. In addition to literature found via PubMed, data publicly available on websites of the World Health Organization (WHO), the United Nations Food and Agriculture Organization (FAO), United States Centre of Disease Communication (US CDC), International Livestock Research Institute (ILRI), Ethiopia’s One Health responsible Ministries and other websites were also included.

## General perspective of One Health

### Definition of One Health

**Fig. 1:**
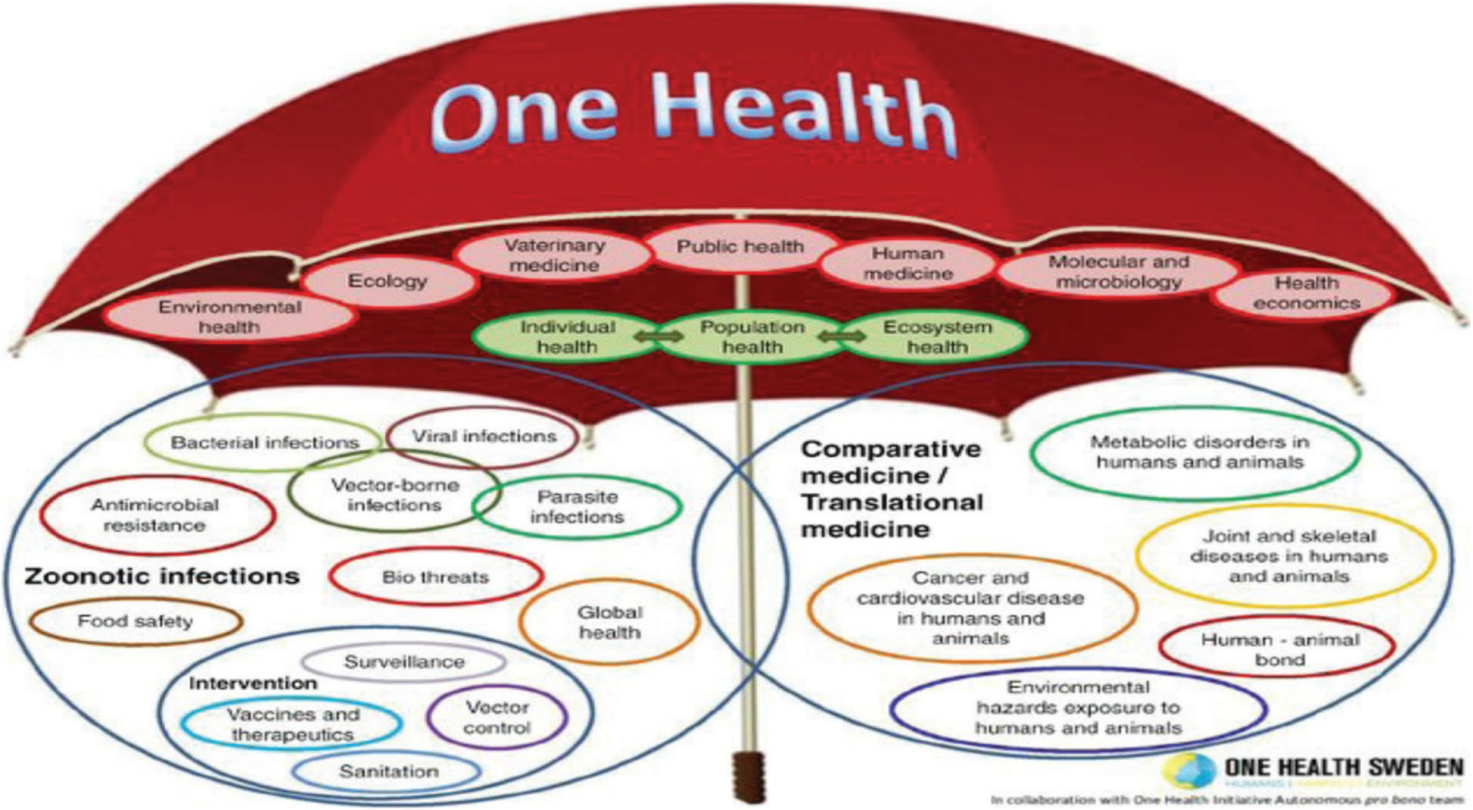
The ‘One Health Umbrella’ developed by the networks ‘One Health Sweden’ and ‘One Health Initiative’ to illustrate the scope of the ‘One Health concept’ [17]

The terms ‘One Medicine’ and ‘One Health’ have been used to describe the concept of an integrated approach to animal, human and environmental health and to acknowledge that we are all part of ‘One World’ in which animals, people and the environment are interdependent and must rely on each other for basic survival [12].One Health is collaborative approach for strengthening systems to prevent, prepare, detect, respond to, and recover from infectious diseases and related issues such as antimicrobial resistance that threatens human -animal—environmental health collectively [1]. It is an approach as ‘a collaborative and all-encompassing way to address animal and public health globally not only at international level, but must be translated as a new paradigm at national levels’[13]. One Health approach is described as either a narrow approach primarily combining public health and veterinary medicine or as a wide approach as in the wide-spread ‘umbrella’ depiction (Fig. 1) including both scientific fields and interdisciplinary research areas [14], 15. The One Health concept is a worldwide strategy for expanding interdisciplinary collaborations and communications in all aspects of health care for humans, animals and the environment. The synergism achieved will advance health care for the twenty-first century and beyond by accelerating biomedical research discoveries, enhancing public health efficacy, expanding scientific knowledge, and improving medical education and clinical care. When properly implemented, it will help protect and save untold millions of lives in our present and future generations [16].

**Fig. 2:**
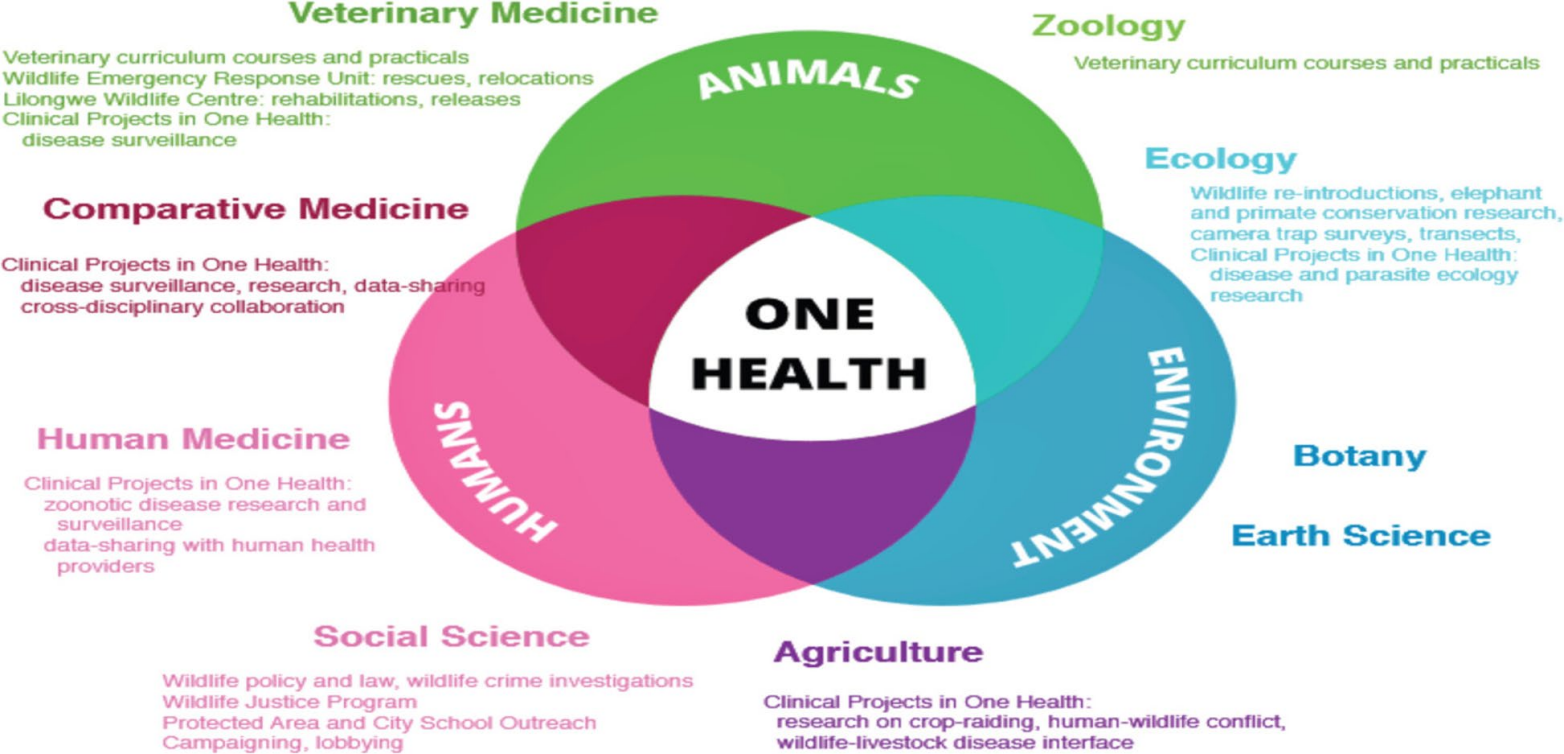
Typical example of one health coordination, Lilongwe Wildlife Trust, Clinical Project in One Health, Malawi [19]

One Health is a collaborative, multi-sectoral, and trans-disciplinary approach (Fig. 2)—working at the local, regional, national, and global levels—with the goal of achieving optimal health outcomes recognizing the interconnection between people, animals, plants, and their shared environment [18].

### The rise of One Health concept

Rudolf Virchow (1821–1902) considered as the father of comparative medicine, cellular biology and veterinary pathology for his contribution to medicine, incorporating veterinary medicine in human health care and effectively launched the One Health concept in the nineteenth century. He asserted that there is no dividing line nor should there be between animal and human medicine. He also coined the term “zoonosis” for a disease transmissible from animals to humans. This founding concept is a worldwide paradigm shift strategy for expanding interdisciplinary collaborations and communications in all aspects of health care for humans and animals [20]. The building concept of “**One World, One Health**” was also formulated by the Wildlife Conservation Society in 2004 by establishing an interdisciplinary and cross-sectoral approach to prevent epidemic or epizootic disease and for maintaining ecosystem integrity [21]. In 2008, the importance of this concept is further strengthened by the FAO, OIE,WHO, United Nations Children’s Fund), the World Bank and United Nations System of Influenza Coordinator and produced a document entitled **‘contributing to One World, One Health’,** a strategic framework for reducing risks of infectious diseases at the animal-human-ecosystems interface [22]. The World Medical Association (WMA) in its resolution on the collaboration between Human and Veterinary Medicine, adopted in October 2008, recommends the collaboration between human and veterinary medicine and supports the concept of joint educational efforts between human and veterinary medical schools [21]. Since then the One Health concept has become more important and in recent years its initiatives have been rapidly gaining ground [23]. Another important action, which increased the platform of One Health, is the Global Conference on One Health (held in Spain, May 2015) by the World Veterinary Association (WVA) and the World Medical Association (WMA),. The conference has recommended the need to increase cross-disciplinary collaboration between the veterinary and medical professionals in order to improve human and animal well-being [24].

### Global One Health initiatives

The importance and interventions of One Health are fundamentally linked in food systems, health impact of zoonotic diseases, drug resistance, economic losses and many other health impacts [5]. The risk of spreading emerging and reemerging diseases has been increasing by the interactions of human– animal–ecosystem increases due to the exponential growth in human and livestock populations, rapid urbanization, rapidly changing farming systems, integration between livestock and wildlife, forest encroachment, destruction of habitat, changes in ecosystems, and the globalization of trade [2]. Due to this, there is a need for interconnections among the health of humans, animals, and the environment for effective prevention and control measures through collaboration approach [25]. Hence, several institutions are formally supporting One Health approaches in the country and at global level [24]. With national, transnational or global partnerships, various One Health collaboration actions have achieved so far, such as controlling rabies in Bali, Indonesia; controlling Q fever outbreaks in the Netherlands; the Human Animal Infections and Risk Surveillance (HAIRS) group in the United Kingdom; control of food borne *Salmonella* in the European Union [26]. The WHO, OIE, and FAO also formed the **tripartite** agreement in 2010 to work together on AMR, rabies, zoonotic influenza, zoonotic tuberculosis and Middle East Respiratory Syndrome-Coronavirus (MERS-CoV) [27]. A One Health Operational Framework published by the World Bank in 2018 also provides an overview and compendium of work with key initiatives and entities, implementation guidance, and an annex of resources [1]. Several agencies in various countries have One Health webpages with a variety of resources, including US CDC [28]. The WHO, FAO and OIE, as well as the United Nations Environmental Programme (UNEP) are working together to facilitate cross-sectoral collaboration at the global level to manage health risks and improve global health security [29].

Currently, FAO and WHO through the **Global Health Security Agenda’s** Zoonotic Diseases and Animal Health in Africa (GHSA-ZDAH) has been supporting many One Health interventions through policy documents, control strategies, protocols, evaluations, national veterinary laboratories strengthening, epidemio-surveillance capacity and workforce development [30]. The One Health approach has been adopted by various countries as the core driver of the Global Health Security Agenda (GHSA) [31]. The Global Health Security Agenda (GHSA), an alliance of over 70 governments and international partners, was launched in February 2014 with the aims of driving and advocating for a world safe and secure from infectious disease threats; bringing together nations from all over the world to make new, concrete commitments, and elevating global health security as a national leaders-level priority [32]. Due to its urgency and various importance, One Health approach has increasingly been adopted in national and international plans and strategies of many countries [33].

## One Health frameworks in Ethiopia

Ethiopia is a GHSA member country and many of its One Health activities are supported within the framework of improving global health security. While emerging and epidemic-prone diseases such as avian influenza and Ebola present a global threat, endemic zoonotic diseases, such as rabies and anthrax, affect the health of animals and humans and are a major source of economic loss [10]. In recognition of the intrinsic relationship between humans, animals, and their environment, and as part of the implementation of the GHSA, the country increasingly has embraced the One Health approach to prevent, detect, and respond to existing and emerging threats and there is a strong political commitment by the government [34], 14.

### The establishment of One Health platform

In 2015, the first One Health Zoonotic Disease Prioritization workshop, in Ethiopia, was held and brought multiple ministries and partners together to develop a list of zoonotic diseases of the greatest national concern. Key national government stakeholders involved and implemented joint zoonotic disease surveillance, control and outbreak response activities such as such as MOH, MOA) and the EWCA were involved in the implementation of joint zoonotic disease surveillance, control and outbreak response activities [34]. Within the MOH, the EPHI leads human outbreak investigations, surveillance, and laboratory diagnostics for humans, as well as diagnostics for rabies in animals. Within the MOA, the Veterinary Public Health Directorate, the Disease Prevention and Control Directorate, and the Epidemiology Directorate are primarily responsible for surveillance and response activities in livestock, in partnership with NAHDIC), where most animal disease diagnostics are performed. The EWCA is responsible for the conservation and management of wildlife and its habitats in collaboration with MOA. In line with this, One Health collaborations have continued and the National One Health Steering Committee (NOHSC) was established and got momentumin its effort to create a sustainable national One Health Platform [10].

### The development of National One Health Steering Committee and signing of memorandum of understanding

**Fig. 3:**
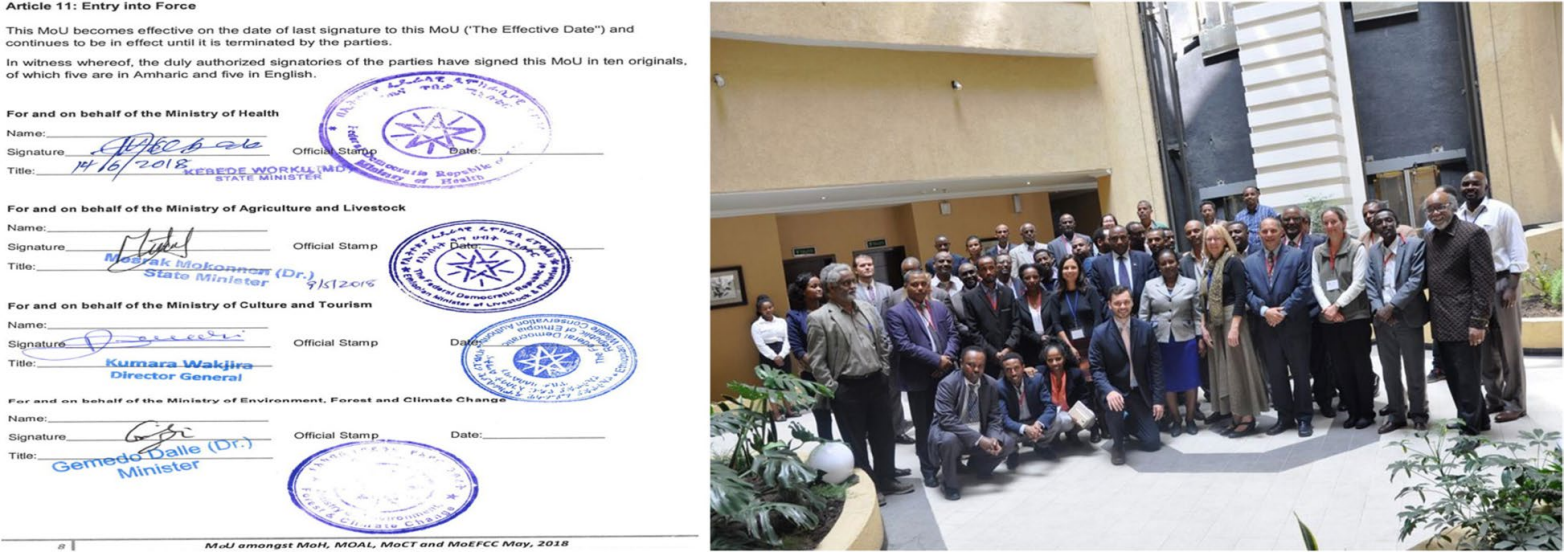
The founding members of Ethiopian National One Health Steering Committee and the Signed Memorandum of Understanding (obtained from NOHSC with permission)

In 2016, four key Ethiopian Ministries joined together to establish the NOHSC with the support of the government of Ethiopia and other partners [35]. The members of the NOHSC were constituted from the MOH, MOA, EWCA, MCT and Universities such as Addis Ababa, Jimma and Mekelle representing OHCEA. In addition, International Organizations and Partners participated in NOHSC include: US CDC, FAO, USAID, WHO, Emerging Pandemic Threat (EPT-2) Partners, and OSU-GOHI). Only government stakeholders had a voting right during the period of the establishment. The Steering Committee has an operational framework of a chair, co-chair and secretary nominated from core government sectors. The committee would hold a meeting on a monthly basis. It shall also adopt a formal system of recording its business in line with relevant rules and guidelines from the government of Ethiopia [10]. One of the NOHSC’s primary goals is to strengthen zoonotic disease prevention, detection and response through a long-term and collaboration at the national and sub-national level. The responsibilities of the Committee’s leadership are to ensure balance and overcome previous problems with multi-sectoral coordination as well as promoting one health goal. The Steering Committee received financial and technical support from partners, local non-governmental organizations (NGOs); and Ethiopian universities [36]. The NOHSC has laid many critical milestones since its inception. For example, the four Ethiopian Ministries signed the memorandum of understanding (MOU) (Fig. 3) in 2018 which was believed to formalize the commitment between the parties to work together on joint disease surveillance, data sharing, preparedness and communication planning, outbreak investigation and response, and related activities. It has also drafted a National One Health Strategic Plan for 2018–2022. This strategic plan included an organizational framework with detailed guidance on how the National Steering Committee would address One Health engagement across disciplines and sectors in its task to prevent, detect and respond to endemic, emerging and re-emerging infectious disease threats at the human–animal–environment interface [34].

### Initiatives which encourage ethiopian One Health approach

There are various Partner One Health Initiatives in the country which have been working in collaborations with government and other institutions, such as universities/research institutions, Non-Governmental Organizations (NGOs) and donor organizations with three main purposes: the prevention of new zoonotic disease emergence, early detection of new threats, and timely and effective response to them [35]. According to Onyango et al. [36] and Fasina and Fasanmi [14]some of them include:

- Jigjiga One Health Initiative (JOHI) is funded by the Swiss Agency for Development and Cooperation (SDC) and run by Jigjiga University, the Armauer Hansen Research Institute (AHR) and the Swiss Tropical and Public Health Institute in Basel. It was aimed at building the capacity of Jigjiga University to become a center of excellence for One Health studies and creating innovative systems for the improvement of health and wellbeing of pastoral communities.
- The Ohio Global One Health Initiative by the Ohio State University Health Sciences which focused on improving the capacity of pre-service health professionals in Ethiopia and established the African regional office in Addis Ababa in 2017.
- One Health Regional Network For the Horn Of Africa is a multidisciplinary, international partnership led by the University of Liverpool in partnership with Liverpool School of Tropical Medicine, United Kingdom; University of Nairobi, and International Livestock Research Institute, Kenya; University of Addis Ababa, and the International Livestock Research Institute, Ethiopia; Sheikh Technical Veterinary School, Somaliland; Hamelmalo Agricultural College, Eritrea; and other national and international organizations. The project was funded by the Biotechnology and Biological Sciences Research Council Fund and aims at improving the research capacities of individuals and institutions particularly on human and animal health issues and creating a One Health Regional Network for knowledge and information sharing [14].
- One Health Central and East African (OHCEA) University Network is a network of 21 public health and veterinary universities from 8 countries in the East, Central and West Africa regions. In Ethiopia, 3 universities, Jimma, Mekelle and Addis Ababa are members of this network that aims at cultivating the culture of multi-sectoral collaboration through field attachment, experiential learning, training and research.
- Moreover, international organizations such as USAID, WHO, CDC, FAO and others have been aggressively supporting one health issues and working on advocacy and awareness on antimicrobial resistance.

## One Health approach achievements in Ethiopia

### The establishment of different technical working groups

The National One Health Steering Committee has established different national Technical Working Groups (TWGs) including Rabies, Anthrax, Brucellosis, Emerging Pandemic Threats (EPT), Antimicrobial Resistance (AMR) and National One Health Communication Task Force to promote multi-sectoral coordination and collaboration on One Health related activities. Each TWG represents a specific zoonotic disease with particular emphasis on prioritized ones (such as anthrax, rabies and brucellosis) and main pandemic threats like highly pathogenic avian influenza, Rift Valley Fever (RVF). The technical working groups are composed of veterinary and medical experts in virology, bacteriology, microbiology and epidemiology and provide a platform for strategic discussions. The working group members include government and non-government stakeholders and are officially nominated from line ministries to the National One Health Steering Committee [10].

### Extension of One Health schemes to the regional governments

So far, the national One Health coordination structures have already been extended to 7 Regions (Amhara, Oromia, Southern Nations Nationalities People Region (SNNPR), Tigray, Somali, Benishangul-Gumuz, and Gambella). In addition to this, the structure has further extended to 7 Zones, and 17 districts in different regions of the country [37].

### The development of national One Health strategic plan (2018 -2022)

**Fig. 4:**
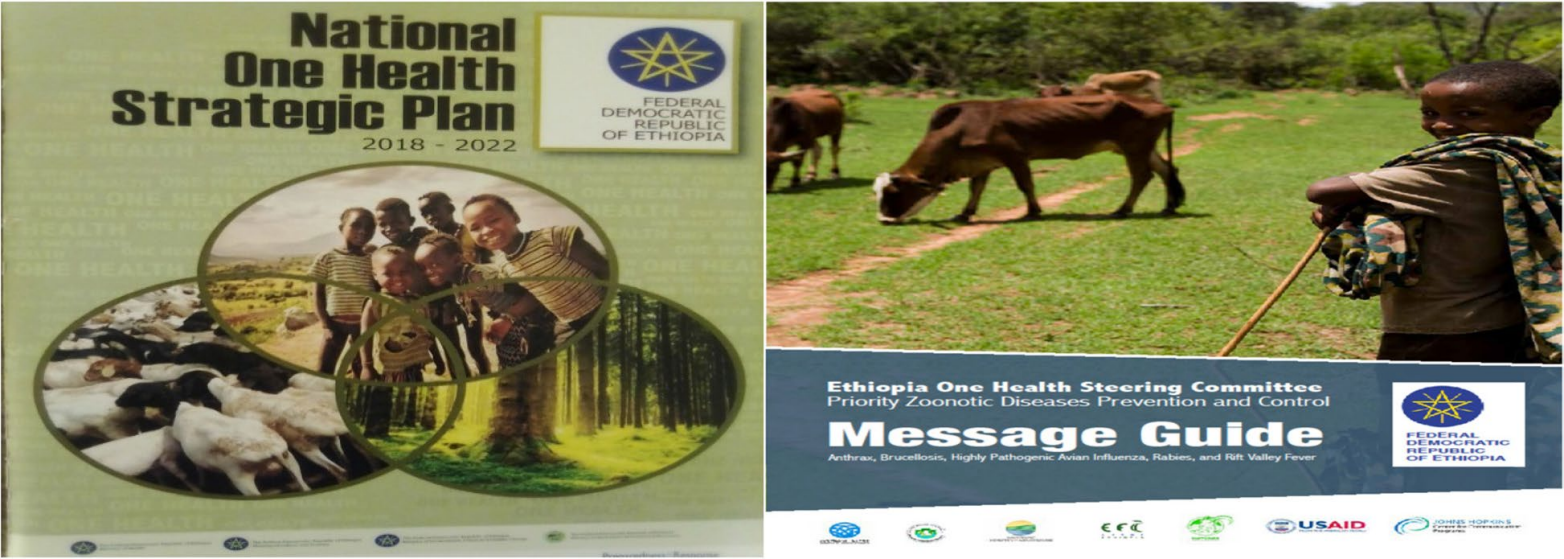
The National One Health Strategic Plan and Its Communication Message Guide taken from NOHSC with permission)

The NOHSC developed a National One Health Strategic Plan (2018–2022) for the overall guidance of one health approach in Ethiopia (Fig. 4). The strategic plan is the roadmap for the country to achieve the long-term goal of prevention, detection and response to “negligible risks and impacts of endemic, emerging and re-emerging health threats at the animal-environment-human interface". Moreover, the strategic plan includes an organizational framework with detailed guidance on how the NOHSC will address One Health engagement across disciplines and sectors in its tasks [38]. Ethiopia aims to achieve this goal through the five key pillars and objectives which include: coordination and collaboration to ensure effective one health schemes, preparedness and response to emerging and re-emerging priority threats, multi-sectoral surveillance and reporting system, advocacy and communication as well as research and capacity building. The National One Health Steering Committee has also developed and been implemented a Risk Communication and Community Engagement (RCCE) strategy document (Fig. 4) which provide a comprehensive guidance for response zoonotic disease including COVID-19 outbreak and to mitigate the impact of emerging and reemerging diseases,

### The development of control and prevention strategic documents for different prioritized zoonotic diseases

Several strategic documents for prioritized zoonotic diseases have also been developed and validated by each National Technical Working Groups together with partners and other stakeholders and finally endorsed by the National One Health Steering Committee. With the overall leadership of the members of each National Technical Working Group in drafting their strategic document, key responsible ministries, regional health and livestock and/or animal health bureaus, research institutions, universities and development partners have engaged during the development of the document. Each strategic document is a joint plan of key ministries ( Ministry of Health, Ministry of Agriculture, Ministry of Culture and Tourism and Ministry of Environment, Forest and Climate Change which has been endorsed by NOHSC. International partners and other stalk holders were also represented and actively participated during the development and validation of the documents. Each strategic document has its own framework (developed based on OIE, WHO, FAO principles), stepwise approach and implementation phases.

**Fig. 5:**
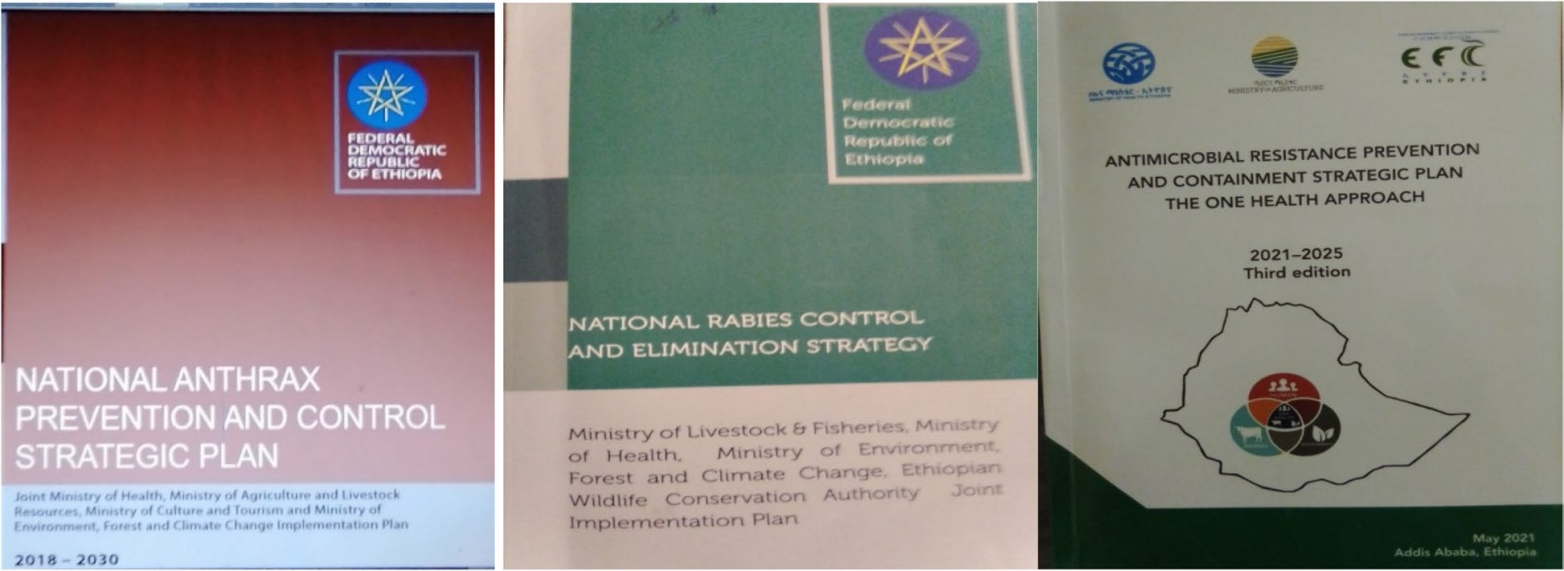
Some of validated One Health approach strategic documents (obtained from Ministry of Agriculture with permission)

The first national control and prevention strategy document which has been endorsed to be implemented from 2018–2030 and currently under enactment is the National Rabies Control and Elimination Strategic Document. It was developed with the goal to eliminate all human rabies deaths by 2030 through a strategic vaccination campaign that achieves and maintains a vaccination rate of at least 70% of the domestic dog population in the country (Fig. 5). The second strategic document which was endorsed to be implemented from 2018 – 2030 is Anthrax Prevention and Control Strategic Plan. The overall mission of this plan is to significantly reduce and ultimately control the public health impact of anthrax in humans and animals, in Ethiopia, through sustained surveillance, laboratory diagnosis, prevention and control systems and community awareness (Fig. 4).The other strategy document is the National Brucellosis Prevention and Control Strategic Plan (2020–2030). This has the mission of reducing the impact of brucellosis in livestock and humans in Ethiopia by 2030 through multi-sectoral and community engagement at all levels.

The fourth important strategic documents, prepared by the key ministries having a role in the one health activities, are a Multi-sectoral Preparedness and Response Plan for Highly Pathogenic Avian Influenza. It was prepared with financial support of Partners. The purpose of the preparedness and response plan is to prevent and/or mitigate transmission of pandemic Avian Influenza virus strain and protect the health, social and economic wellbeing of the population. Rift Valley Fever Multi-sectoral Preparedness and Response Plan is also among the strategic documents. The plan is thought to address prevention and control of Rift Valley Fever in humans and animals through professionals and relevant institutions through involvement of professionals in the surveillance, detection and response to RVF outbreaks. The scope could extend to bilateral agreements with neighboring countries to jointly prevent and control the threat. Another important strategic document is Prevention and Containment of Antimicrobial Resistance. Its goal is to prevent, slow down, and contain the spread of antimicrobial resistance through the continuous availability of safe, effective, and quality-assured antimicrobials and their effective use thereof. This can only be achieved through collaborative actions among partners in human health, animal health, the environment, agriculture, the food industry, teaching and research institutes, civil societies and associations, the pharmaceutical industry, and global stakeholders to synergize efforts and resources (Fig. 5).

### Joint disease surveillance and outbreak investigation activities

**Fig. 6:**
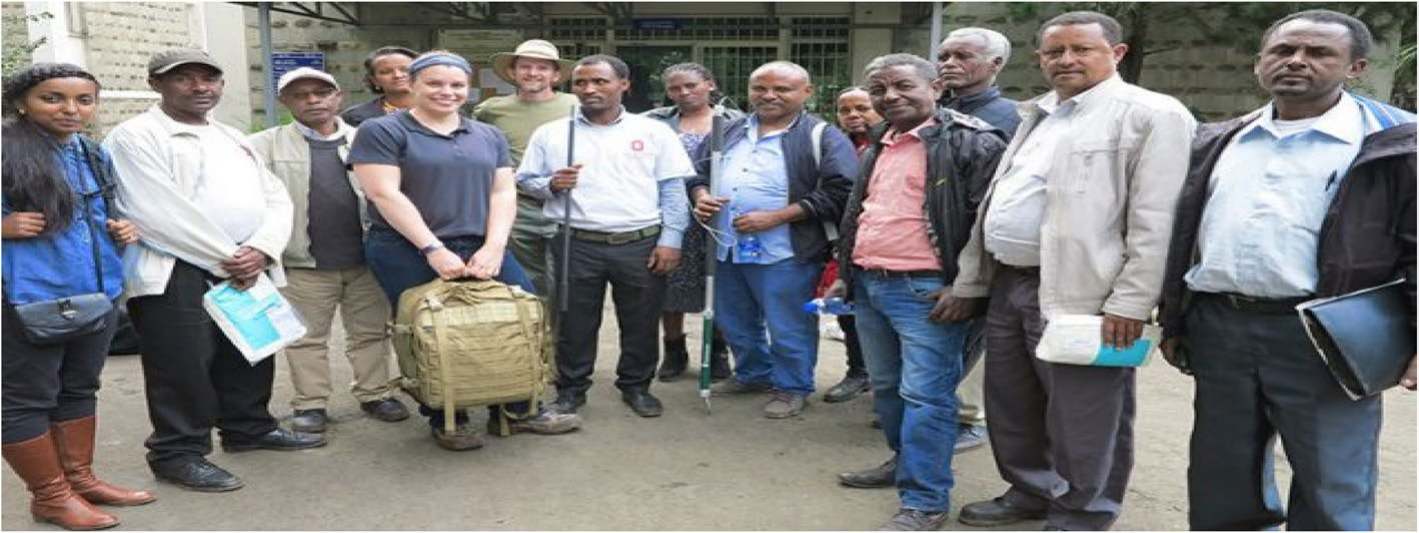
CDC and Ethiopian animal rabies surveillance officers in Ethiopia, 2016 [39]

The country with the Technical Working Groups (TWGs) has been coordinating and conducting joint disease surveillance and outbreak investigations activities following reports received from various locations in the territory of the country. It has conducted joint anthrax disease outbreak investigations in 2018 for suspected animal and human cases. The investigation team consisted of regional veterinarians, medical workers and national-level laboratory and epidemiology experts were deployed for the investigation. Safe sample collection and transportation training was completed only days before the outbreak investigation mentioned above. As a result, the responders were better prepared and equipped to collect samples from both animal and human suspected cases (Fig. 6). A joint investigation team composed of community animal and human health experts, local representatives and faculty members of College of Health sciences of Jimma University were engaged on another anthrax outbreak investigation in Oromia Regional State in 2018. In the same year, Rabies joint outbreak investigation was led by the Ministry of Health and Ministry of Agriculture after having trained on animal sample collection and transportation. Furthermore, in the mid-2018, Rift Valley Fever outbreak was reported across an extensive geographic range in East Africa, including areas bordering Ethiopia. Thus, in preparation for its possible spread to Ethiopia, the Ministry of Agriculture worked with the NOHSC to organize One Health preparedness planning, coordinating teams to conduct enhanced surveillance activities in at-risk border zones [10].

**Fig. 7:**
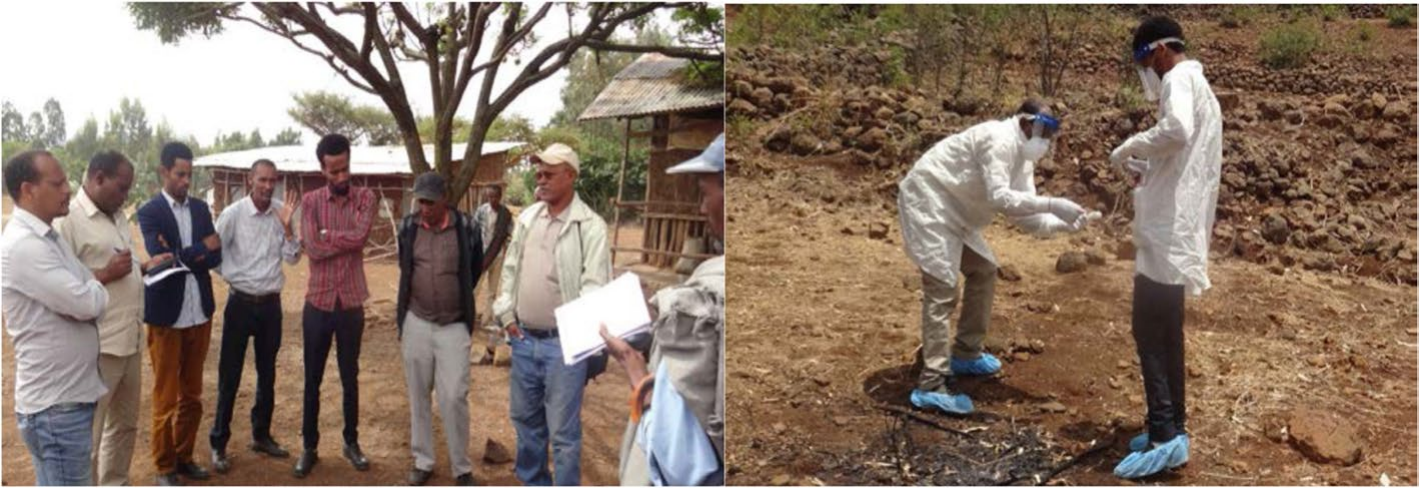
Joint outbreak field investigation team discussing while anthrax outbreak investigation and Laboratory workers collect samples from the bone remains of a suspected anthrax case [10]

A multidisciplinary and multi-sectoral team (Fig. 7). was also organized and deployed by the Emergency Pandemic Threat-Technical Working Group for joint survey and potential outbreak investigation was conducted in the Borena zone of Oromia region. This was conducted following the Rift Valley Fever outbreak report in northern Kenya and mass wild birds (pigeons) mortality in the South Omo zone of Southern Nations Nationalities People Region. This multidisciplinary team also conducted anthrax outbreak investigation in Wag Himra and North Gondar zones of Amhara Region and Assosa zone of Benishangul-Gumuz region.

### Vaccination activities against zoonotic diseases

The Ministry of Health represented by the Ethiopian Public Health Institute (EPHI) and Ministry of Agriculture with the support of global partners US CDC, Ohio State University Global One Health Initiative (GoHi) and the Global Alliance for Rabies Control, and the European Union-Health of Ethiopian Animal for Rural Development (HEARD) project have been conducting mass dog vaccination campaign(MDVC) in collaboration with regional and city administrations. Since 2016, more than 50,000 dogs have been vaccinated. The campaigns were achieved after providing training for veterinary, medical and public health staff regarding animal handling, vaccine safety, vaccination evaluation and dog population estimation methods. The vaccination activities were a reflection of how successful One Health collaborations among government partners were. It also showed how strategic support and mentoring from global experts can help in materialising and sustaining the goals of the TWGs and workforce [10].

### Prioritization of zoonotic diseases in Ethiopia

There are three strategies — predict, respond, and prevent — and eleven packages which were developed to achieve the strategies by GHSA. One of the main packages is addressing the burden of zoonotic diseases [40]. In Ethiopia, there are large numbers of zoonotic diseases which are endemic. Hence, prioritization of zoonotic diseases based on impacts on both human and animal is of paramount importance so as to jointly address experts from both animal health agencies and public health authorities. Accordingly, two prioritization processes of zoonotic diseases were conducted; in 2016 and 2019.

### The first prioritization of zoonotic diseases in Ethiopia

The first zoonotic diseases prioritization workshop in Ethiopia was held in 2015 by participating organizations form Federal Ministry of Health represented by Ethiopian Public Health Institute, the Ministry of Livestock and Fishery Resources, the Ministry of Environment and Forestry, WHO, United States Centres for Disease Control and Prevention, Defence Threat Reduction Agency/Cooperative Biological Engagement Program, the Ohio State University, Food and Agriculture Organization of the United Nations, and Armauer Hansen Research Institute/Swiss Tropical and Public Health Institute using a in Addis Ababa [7]. The workshop participants identified five criteria for ranking among 43 zoonotic diseases through group discussion. The criteria used to select the final five prioritized zoonotic diseases are unique to Ethiopia and includes: Severity of human disease in Ethiopia (diseases having the highest number of deaths rates per population in humans were deemed to have priority), proportion of human disease attributed to animal exposure, burden of animal disease (priority was given to diseases that have negative impacts at the household level in Ethiopia by causing production losses in Livestock), availability of interventions (vaccines targeting diseases in animal and medical intervention available for people), and existing inter-sectoral collaboration ( disease which has focus of inter-sectoral collaboration gained full credit). Finally, five top zoonotic diseases (rabies, anthrax, brucellosis, leptospirosis, and echinococcosis) were selected and ranked for inter-sectoral engagement by human and animal health agencies [39].

### Re-prioritization of zoonotic diseases in Ethiopia

**Fig. 8:**
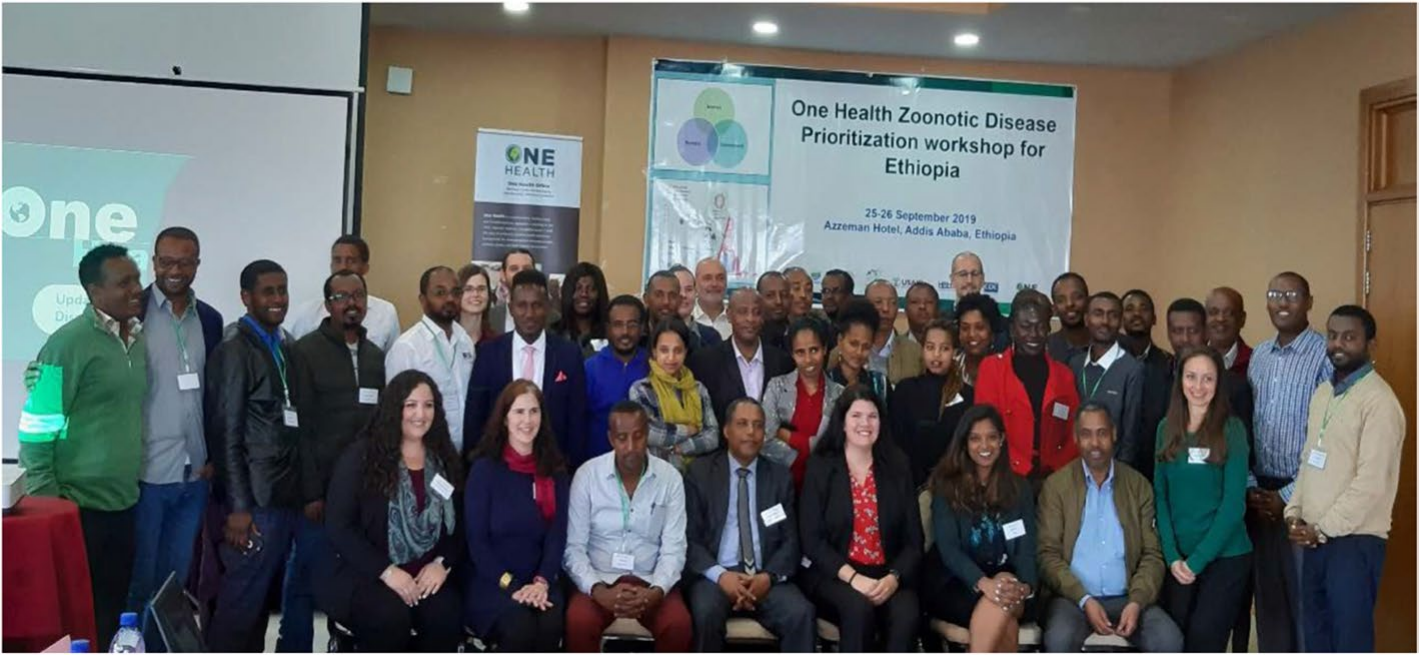
Participants of One Health zoonotic disease re-prioritization workshop in Ethiopia, Photo taken from the workshop by the first author

Ethiopia is the first country in Africa to utilize the One Health Zoonotic Disease Prioritization Process (OHZDP) for the second time to update the priority zoonotic disease list. Because of the request from Ministry of Agriculture and other relevant stakeholders for the reprioritization of current country’s public health and economic importance of diseases, the National One Health Steering Committee in collaboration with US CDC and Human Resource for Health -2030 (HRH2-030) organized national level zoonotic diseases re-prioritization workshop from September 24–25, 2019 in Addis Ababa. Experts from national and regional level and key stakeholders of the National One Health Steering Committee (Ministry of Health; Ministry of Agriculture; Environment, Forestry, and Climate Change Commission; and the Ethiopian Wildlife Conservation Authority and partners such as USAID, CDC, FAO, WHO, Veterinary Sans Frontiers -Suisse, etc.…) were participated on the workshop (Fig. 8).. Accordingly, 41 Zoonotic diseases were considered for prioritization and criteria utilized to determine the ranked outcomes of the One Health Zoonotic Disease Reprioritization process are epidemic or pandemic potential, availability of prevention and control strategies, severity in humans, socioeconomic impact and presence of disease in Ethiopia. After the two days exercise, a list of five top zoonotic diseases of greatest national concern was agreed upon by voting members. These are: Anthrax, Rabies, Brucellosis, Rift valley fever, and Zoonotic Avian Influenza which are identified as the five national priority zoonotic diseases in Ethiopia [41].

### Awareness creation

**Fig. 9:**
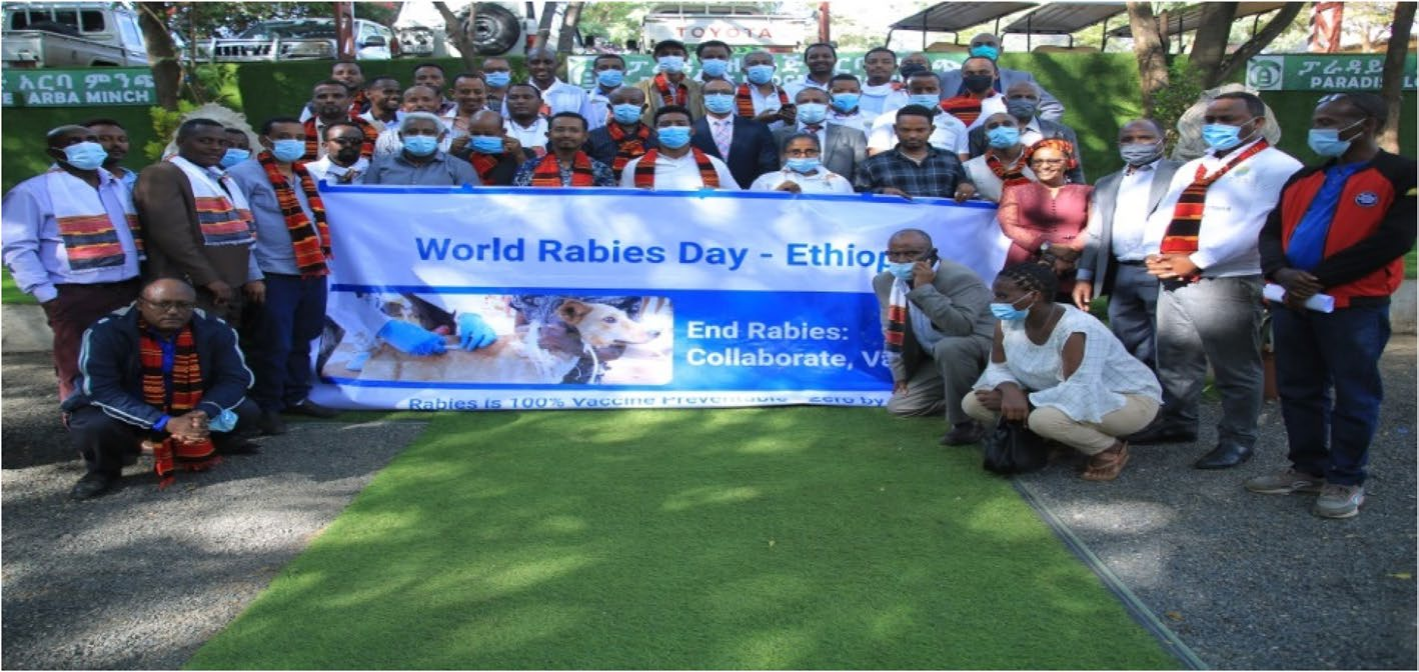
Moment of World Rabies Day Celebration in Ethiopian, 2021 September 28(Ministry of Agriculture)

The NOHSC established National One Health Communication Taskforce (OHCTF) in 2019 to facilitate planning and implementation of advocacy and communication interventions for One Health program in the country. The task force has been working aggressively since its establishment. The communication taskforce has developed One Health website and telegram channel, prepared, printed and distributed many copies of national zoonotic diseases message guide and National Rabies Control and Elimination Strategic Documents (Fig. 4). At the global level, the World Health Organization WHO), OIE, FAO and the Global Alliance for Rabies Control (GARC) have launched 'World Rabies Day' (WRD) campaign in 2007 as a response to the call to raise global awareness and mobilize resources for rabies prevention and control and it has been celebrating annually every September 28 which is the largest unifying initiative on the prevention of the disease [42]. Based on this, celebration of the World Rabies Day in Ethiopia promoting One Health has begun since 2017. The 2021 celebration day workshop was held on 28 September (Fig. 9).

## Opportunities, challenges and solutions of One Health approach in Ethiopia

### Opportunities and challenges

There are several achievements and opportunities to extend the One Health schemes and philosophies to deal with zoonotic diseases in Ethiopia. The opportunities include strong interest from technical people in the ministries, the establishment of the National One Health Steering Committee and prioritized zoonotic diseases Technical Working Groups and their active engagement, and interest and support from various NGOs. However, there are still considerable challenges which stakeholders and responsible government bodies should be aware of [38], 30. According to Fasina and Fasanmi, 2020, some of the challenges include:

- Poor integration among animal and human health sectors in data sharing and lack of awareness and continuous advocacy across the relevant sectors and community members
- Leadership and commitment from higher government officials including budgeting is still not strong
- Weak encouragement and collaboration between regional One health task forces
- Competing priorities among prioritized zoonotic diseases prevention and control strategic plans (Ethiopia has already planned more than three zoonotic diseases to control and eliminate them by 2030).
- Limited laboratory diagnostic capacity, resulted in poor detection of outbreaks/causative agents
- One Health-based course in the curriculum of human medicine, veterinary medicine and other related disciplines in most universities are still not included.
- Lack of clear legislation on the engagement of public–private partnership pertinent to One Health

### Solutions

Possible solutions could be awareness of One Health and foster leaders who are uniquely skilled to work across disciplines and sectors, rapidly Institutionalize the One Health approach. The NOHSC with its TWGs should not only extend the One Health concept to the community level but also begin operation of prioritized zoonotic diseases prevention and control measures based on urgent revision of competing priorities. Universities should include One Health philosophies and principles to academic curricula, including designated degree programs as well as incorporating the One Health research issues into their thematic areas. In addition to these, capacitating diagnostic laboratories, encouraging research activities and advising to increase leadership commitment are very important.

## Conclusion

The risk of spreading of emerging and reemerging zoonotic diseases has been increasing by the interactions of human, animal and ecosystem and accounts for more than a billion cases, a million deaths and hundreds of billions of United States dollars of economic damage per year. The One Health approach is critical for solutions to prevent, prepare for, and respond to these complex threats. Countries, like Ethiopia, in which their household income is dependent on livestock, are characterized by strong correlation between a high burden of zoonotic disease and poverty. Thus, reducing the zoonotic disease burden through OH approach is crucial to improve the overall health. In recognition of the intrinsic relationship between humans, animals, and their environment, and as part of the implementation of the GHSA, the country increasingly has embraced the One Health approach to prevent, detect, and respond to existing and emerging threats. Several One Health initiatives and workforces have been developed so far including the establishment of the NOHSC and different prioritized zoonotic diseases Technical Working Groups, the development of control and prevention strategic documents for different prioritized zoonotic diseases, conducting joint disease surveillance and outbreak investigation activities, re-prioritization of zoonotic diseases**,** beginning of capacity building for diagnostic laboratories and other One Health promotions. Nevertheless, there are still so many challenges which require serious considerations. Poor integration among sectors in data sharing and communication, lack of advocacy, lack of financial support from government, limited research fund and activities etc. are among many challenges. Hence, it is critical to continue to raise awareness of OH and foster leaders who are skilled to work across sectors. Institutionalization of One Health is a vital step in materializing One Health policy. The N OHSC and its different national Technical Working Groups (TWGs) should not only extend the One Health concept to the grassroots level and/or community level but also begin the operation of prioritizing zoonotic diseases prevention and control measures. Universities should include One Health philosophies and governing principles to academic curricula, including designated degree programs as well as incorporating the One Health research issues into their thematic areas.

## Data Availability

All reviewed data and materials during the period of review are included in this article.

## Abbreviations

AMR: Antimicrobial resistance
BM: Bedaso Mamo
EPHI: Ethiopian Public Health Institute
EPT: Emerging Pandemic Threat
EU-HEARD: European Union-Health of Ethiopian Animal for Rural Development
EWCA: Ethiopian Wildlife Conservation Authority
FDRE: Federal Democratic Republic of Ethiopia
FR: Fikru Regassa
GA: Gashaw Adane
GM: Gezahegne Mamo
GARC: Global Alliance for Rabies Control
GHSA: Global Health Security Agenda
ILRI: International Livestock Research Institute
MCT: Ministry of Culture and Tourism
MDVC: Mass Dog Vaccination Campaign
MERS-CoV: Middle East Respiratory Syndrome-Coronavirus
MOA: Ministry of Agriculture
MOH: Ministry of Health
MOU: Memorandum of Understanding
NAHDIC: National Animal Health Diagnostic and Investigation Center
NOHSC: National One Health Steering Committee
OHA: One Health Approach z
OHCEA: One Health Central and Eastern Africa
OHCTF: One Health Communication Taskforce
OSU-GOHI: Ohio State University-Global One Health initiative
SNNPR: Southern Nations Nationalities People Region
TWG: Technical Working Groups
UN FAO: United Nations Food and Agriculture Organization
US CDC: United States Centre of Disease Communication
USAID: United States Agency for International Development
WHO: World Health Organization
WRD: World Rabies Day

## References

[1] Franck Cesar Jean; Bouley Timothy; Karesh WB. LGFG. MCCPCASRM. 2018. B. Operational framework for strengthening human, animal and environmental public health systems at their interface Washington, D.C. : World Bank Group. [Internet]. 2018. Available from: https://documents.worldbank.org/en/publication/documents-reports/documentdetail/703711517234402168/operational-framework-for-strengthening-human-animal-and-environmental-public-health-systems-at-their-interface

[2] Jones BA, Grace D, Kock R, Alonso S, Rushton J, Said MY (2013). Zoonosis emergence linked to agricultural intensification and environmental change. Proc Natl Acad Sci U S A.

[3] Karesh WB, Dobson A, Lloyd-Smith JO, Lubroth J, Dixon MA, Bennett M (2012). Ecology of zoonoses: natural and unnatural histories. Lancet (London, England).

[4] Bennett JE, Stevens GA, Mathers CD, Bonita R, Rehm J, Kruk ME, et al. NCD Countdown 2030: worldwide trends in non-communicable disease mortality and progress towards Sustainable Development Goal target 3.4. Vol. 392, The Lancet. Lancet Publishing Group; 2018. p. 1072–88. 10.1016/S0140-6736(18)31992-5 30264707

[5] Machalaba CC, Salerno RH, Barton Behravesh C, Benigno S, Berthe FCJ, Chungong S (2018). Institutionalizing One Health: From Assessment to Action. Heal Secur.

[6] McDermott JJGD. Agriculture-associated diseases: Adapting agriculture to improve human health [Internet]. 2012 [cited 2022 Feb 7]. Available from: https://cgspace.cgiar.org/handle/10568/16449

[7] Pieracci EG, Hall AJ, Gharpure R, Haile A, Walelign E, Deressa A (2016). Prioritizing zoonotic diseases in Ethiopia using a one health approach. One Heal (Amsterdam, Netherlands).

[8] CDC. History of Ebola Virus Disease (EVD) Outbreaks [Internet]. 2022. Available from: https://www.cdc.gov/vhf/ebola/history/chronology.html.

[9] Africa CDC. Alert Notification: Highly Pathogenic Avian Influenza (HPAI) H5N8 – Africa CDC [Internet]. 2020 [cited 2022 Feb 3]. Available from: https://africacdc.org/download/highly-pathogenic-avian-influenza-hpai-h5n8-notification/

[10] Murphy SC, Negron ME, Pieracci EG, Deressa A, Bekele W, Regassa F (2019). One Health collaborations for zoonotic disease control in Ethiopia. Rev Sci Tech.

[11] Comitato Collaborazione Medica ICVSF. ONE HEALTH POLICY CONTEXT OF ETHIOPIA, SOMALIA, AND KENYA available https://www.google.com/search?client=firefox-b-d&q=Ethiopia National One Health Strategic Plan %282018+%E2%80%93+2022%29++#. 2019.

[12] Osburn B, Scott C, Gibbs P (2009). One world–one medicine–one health: emerging veterinary challenges and opportunities. Rev Sci Tech.

[13] OIE. One Health [Internet]. 2014 [cited 2021 Nov 24]. Available from: https://www.oie.int/en/what-we-do/global-initiatives/one-health/

[14] Fasina and Fasanmi OG. The One Health landscape in sub-Saharan African countries. Nairobi, Kenya: ILRI. available at https://www.ilri.org. 2020. 10.1016/j.onehlt.2021.100325 PMC8455361 34584927

[15] Lerner H, Berg C (2015). The concept of health in One Health and some practical implications for research and education: what is One Health?. Infect Ecol Epidemiol.

[16] OHI. One Health Initiative [Internet]. 2020 [cited 2021 Nov 26]. Available from: https://onehealthinitiative.com/

[17] Gibbs EPJ. The evolution of One Health: a decade of progress and challenges for the future. Vet Rec [Internet]. 2014 Jan 1 [cited 2021 Nov 23];174(4):85–91. Available from: https://onlinelibrary.wiley.com/doi/full/10.1136/vr.g143 10.1136/vr.g143 24464377

[18] CDC. One Health Basics [Internet]. 2018 [cited 2021 Nov 26]. Available from: https://www.cdc.gov/onehealth/basics/index.html

[19] LWT. Clinical Projects in One Health,Malawi [Internet]. 2019 [cited 2021 Nov 26]. Available from: https://www.lilongwewildlife.org/clinical-project-one-health/

[20] Monath TP, Kahn LH, Kaplan B (2010). Introduction: one health perspective. ILAR J.

[21] Buttigieg M (2015). A review of the One Health concept: increasing awareness and collaboration between the Maltese medical and veterinary professionals. Malta Med J.

[22] Coleman W (2008). Contributing to One World. One Health. Med Hist.

[23] Jones KE, Patel NG, Levy MA, Storeygard A, Balk D, Gittleman JL (2008). Global trends in emerging infectious diseases. Nature..

[24] WHO and SCBD. Connecting Global Priorities: Biodiversity and Human Health. WHO Press [Internet]. 2015;(June 2017):364. Available from: https://www.cbd.int/health/SOK-biodiversity-en.pdf

[25] Kelly TR, Machalaba C, Karesh WB, Crook PZ, Gilardi K, Nziza J (2020). Implementing One Health approaches to confront emerging and re-emerging zoonotic disease threats lessons from PREDICT. One Heal Outlook..

[26] Vandersmissen A, Welburn SC (2014). Current initiatives in One Health: consolidating the One Health Global Network. Rev Sci Tech.

[27] WHO/OIE/FAO. Taking a Multisectoral One Health Approach : A Tripartite Guide to Addressing Zoonotic Diseases in Countries [Internet]. 2019. Available from: http://www.fao.org/documents/card/en/c/CA2942EN/

[28] WHO. Global Health at the Human-Animal-Ecosystem Interface [Internet]. 2020 [cited 2021 Nov 24]. Available from: https://knowledge.unccd.int/cbm/global-health-human-animal-ecosystem-interface

[29] WHO. Zoonoses [Internet]. 2020. Available from: https://www.who.int/news-room/fact-sheets/detail/zoonoses

[30] Fasina FO, Fasanmi OG. The One Health landscape in sub-Saharan African countries Consumer perception of milk safety in Kenya The One Health landscape in sub-Saharan African countries. 2020;87. Available from: https://www.cgiar.org/funders/10.1016/j.onehlt.2021.100325 PMC8455361 34584927

[31] Munyua PM, Njenga MK, Osoro EM, Onyango CO, Bitek AO, Mwatondo A (2019). Successes and challenges of the One Health approach in Kenya over the last decade. BMC Public Health.

[32] GHSA. Global Health Security Agenda [Internet]. 2020 [cited 2021 Nov 24]. Available from: https://ghsagenda.org/

[33] Machalaba C, Raufman J, Anyamba A, Berrian AM, Berthe FCJ, Gray GC (2021). Applying a One Health Approach in Global Health and Medicine: Enhancing Involvement of Medical Schools and Global Health Centers. Ann Glob Heal.

[34] FAO. The Ethiopia One Health legal framework [Internet]. 2020 [cited 2021 Nov 24]. Available from: https://www.fao.org/documents/card/en/c/ca9490en/

[35] Grace D, Mutua F, Ochungo P, Kruska RL, Jones K, Brierley L, et al. Mapping of poverty and likely zoonoses hotspots Zoonoses Project 4 Report to Department for International Development, UK 2 [Internet]. International Livestock Research Institute; 2012. Available from: https://cgspace.cgiar.org/handle/10568/21161

[36] Dr. Diana Onyango, Micol Fascendini, Dr. Barbara Wieland, Dr. Davis Ikiror DJS and DST. One Health Policy Context of Ethiopia, Somalia and Kenya. 2019;(December):1–40.

[37] HEAL. The One Health Units for Humans, Environment, Animals and Livelihoods project [Internet]. 2019 [cited 2021 Nov 24]. Available from: https://www.oh4heal.org/

[38] Grace, Florence Mutua, Pamela Ochungo, Russ Kruska, Kate Jones, Liam Brierley, et al. Mapping of poverty and likely zoonoses hotspots Zoonoses Project 4 Report to Department for International Development, UK [Internet]. Report to Department for International Development, UK. International Livestock Research Institute; 2012 Jul [cited 2021 Aug 2]. Available from: https://cgspace.cgiar.org/handle/10568/21161

[39] CDC. Prioritizing Zoonotic Diseases in Ethiopia [Internet]. 2018 [cited 2021 Nov 25]. Available from: https://www.cdc.gov/ncezid/stories-features/global-stories/zoonotic-diseases-ethiopia.html

[40] CDC. Global Health Security Agenda: Action Packages [Internet]. 2020 [cited 2021 Nov 25]. Available from: https://www.cdc.gov/globalhealth/security/actionpackages/default.htm

[41] CDC. Completed OHZDP Workshops | One Health | [Internet]. 2021 [cited 2021 Nov 25]. Available from: https://www.cdc.gov/onehealth/what-we-do/zoonotic-disease-prioritization/completed-workshops.html#ethiopia

[42] WHO. World Rabies Day [Internet]. 2021 [cited 2021 Nov 25]. Available from: https://www.who.int/news-room/events/detail/2021/09/28/default-calendar/world-rabies-day-2021

